# BPC 157 Therapy: Targeting Angiogenesis and Nitric Oxide’s Cytotoxic and Damaging Actions, but Maintaining, Promoting, or Recovering Their Essential Protective Functions. Comment on Józwiak et al. Multifunctionality and Possible Medical Application of the BPC 157 Peptide—Literature and Patent Review. *Pharmaceuticals* 2025, *18*, 185

**DOI:** 10.3390/ph18101450

**Published:** 2025-09-28

**Authors:** Predrag Sikiric, Sven Seiwerth, Anita Skrtic, Mario Staresinic, Sanja Strbe, Antonia Vuksic, Suncana Sikiric, Dinko Bekic, Dragan Soldo, Boris Grizelj, Luka Novosel, Lidija Beketic Oreskovic, Ivana Oreskovic, Mirjana Stupnisek, Alenka Boban Blagaic, Ivan Dobric

**Affiliations:** 1 Department of Pharmacology, School of Medicine, University of Zagreb, 10000 Zagreb, Croatia; 2 Department of Pathology, School of Medicine, University of Zagreb, 10000 Zagreb, Croatia; 3 Department of Diagnostic and Interventional Radiology, University Hospital Centre, 10000 Zagreb, Croatia; 4 Department of Surgery, School of Medicine, University of Zagreb, 10000 Zagreb, Croatia

**Keywords:** BPC 157, angiogenesis, nitric oxide, control/modulation

## Abstract

The healing issue is a central, not completely understood, problem in pharmacology, approached by many concepts. One of the most well-known is Robert’s and Szabo’s concept of cytoprotection, which holds innate cell (epithelial (Robert), endothelial (Szabo)) integrity, protection/maintenance/reestablishing in the stomach to be translated to other organ therapy (cytoprotection→organoprotection) via cytoprotection agent’s effect. Thereby, we defend stable gastric pentadecapeptide BPC 157 therapy, efficacy, pleiotropic beneficial effects along with high safety (LD1 not achieved) against Józwiak and collaborators’ review speculating its negative impact, speculation of angiogenesis toward tumorogenesis, increased NO and eNOS, toward damaging free radicals formation, and neurodegenerative diseases (Parkinson’s disease and Alzheimer’s disease). Contrarily, in wound healing and general healing capabilities as reviewed, as a cytoprotective agent, and native cytoprotection mediator, BPC 157 controls angiogenesis and the NO-system healing functions, and counteracts the pathological presentation of neurodegenerative diseases in acknowledged animal models (i.e., Parkinson’s disease and Alzheimer’s disease), and presents prominent anti-tumor potential, in vivo and in vitro. BPC 157 resolved cornea transparency maintenance, cornea healing “angiogenic privilege” (vs. angiogenesis/neovascularization/tumorogenesis), does not produce corneal neovascularization, but rather opposes it, and per Folkman’s concept, it demonstrates anti-tumor effect in vivo and in vitro. BPC 157 exhibits a distinctive effect on NO-level (increase vs. decrease), always combined with counteraction of free radicals formation, and in mice and rats, BPC 157 therapy counteracts Parkinson’s disease-like and Alzheimer’s disease-like disturbances. Thus, BPC 157 therapy means targeting angiogenesis and NO’s cytotoxic and damaging actions, but maintaining, promoting, or recovering their essential protective functions.

We have found that certain parts of Józwiak and collaborators’ report [1] do not stand (i.e., BPC 157 and probable toxicity, angiogenesis consequences, the role of BPC 157 metabolites, BPC 157 stimulatory effect on nitric oxide (NO)-system), obscuring information on its biological activities, mechanism of action, increasing interest in the BPC 157 peptide, and its application potential. As we reviewed [2], the stable gastric pentadecapeptide BPC 157 is quite distinctive from standard angiogenic peptides in many aspects, as native and stable in human gastric juice for more than 24 h, always given alone has beneficial effects, unmistakably attributed, and clearly established (i.e., results reliable as different application routes (including per-oral) were used in one model) [2,3,4,5,6]. Thereby, surprisingly, these misconceptions were specifically those on BPC 157 pleiotropic beneficial effects and angiogenesis and the NO-system, speculating an unpleasant chain of adverse effects [1] (i.e., carcinoma, neurodegenerative diseases, Table 1). This might be also surprising, given a network of interconnected evidence demonstrated a neurotransmitter-like activity as BPC 157 therapy counteracts in addition to NO-system disturbances, also dopamine, serotonin, glutamate, GABA, adrenalin/noradrenalin, and acetylcholine disturbances, even opposite, whatever specifically related to their receptors, both blockade and over-activity, destruction, depletion, tolerance, sensitization, and channel disturbances counteraction [4,5].

Overall, from the perspectives of angiogenesis and the nitric oxide (NO) system, all criticisms of BPC 157 should be dismissed. This is because the review by Józwiak and colleagues [1] fails to acknowledge that BPC 157 therapy operates outside the framework of the negative aspects of Folkman’s classical angiogenesis model [7,8,9] (Folkman: angiogenic bioactivity of a given molecule→↑cornea neovascularization→↑carcinoma). Moreover, in fact, it may counteract the very mechanisms and pathways that contribute to harmful angiogenesis (i.e., Folkman: bioactivity of a given molecule→↓cornea neovascularization→↓carcinoma). Likewise, it does not fit the profile of a NO-system disruptor, NO-system dysregulation, and a negative chain of events [10].

Thus, the principle is the work of Judah Folkman, who defined the induction of corneal neovascularization and angiogenesis that can lead to tumorigenesis [7,8,9]. In the case of NO-system disturbances as described by Moncada, agents can induce NO-over-activity and over-release, increased free radical formation, and severe disturbances (including neurodegenerative diseases) [10] (see Table 1). We believe that overlooking these essential points is a significant oversight and misrepresentation, leading to BPC 157 being unjustly accused of potentially harmful effects, misleading argumentations, and false interconnections that do not exist. These speculations are exactly opposite to well-established findings reported by previous studies and reviewed in the mentioned reviews [2,3,4,5,6] (Table 1).

Specifically, as reviewed [2,11,12], BPC 157 controls angiogenesis and the NO-system healing functions, and counteracts the pathological presentation of neurodegenerative diseases in acknowledged animal models (i.e., Parkinson’s disease and Alzheimer’s disease) [4,5,13,14], and presents prominent anti-tumor potential, in vivo and in vitro [15,16]. Illustrating the consistency of Parkinson’s disease and Alzheimer’s disease models, there were 1-methyl-4-phenyl-1,2,3,6-tetrahydropyridine (MPTP), reserpine, haloperidol, other neuroleptics, and stroke, as well as tremor, akinesia, catalepsy, mortality, and brain lesion markedly counteracted [4,5]. Note, BPC 157, as a neurotransmitter-like agent, given in reperfusion after bilateral clamping of the common carotid arteries in rats, exhibits beneficial effects in brain lesions, in the nigrostriatal and hippocampal region, in particular, recovery of disabled muscle function, and recovery of disturbed cognitive function [4,5].

Together, the defense of the principles of BPC 157 therapy, effectiveness, and safety [2,3,4,5,6,11,12,13,14,15,16] correspondingly re-elaborate on the consistent beneficial effects of stable gastric pentadecapeptide BPC 157 therapy on angiogenesis and NO-system. First, this is a successful confrontation with invariably persistent corneal ulcers and strong neovascularization, loss of transparency in all control rats. BPC 157 therapy, 2 ng/mL, and 2 µg/mL distilled water, two eye drops/left rat eye immediately after injury induction, and then every 8 h up to 120 h, induces healing of corneal ulcers, maintaining corneal transparency, opposing corneal neovascularization in all BPC 157-treated rats, resolving cornea “angiogenic privilege” [17]. This particular effect as a specific outcome complements BPC 157’ broader therapeutic effects observed in eye disease pharmacology models, i.e., glaucoma, retinal ischemia, see [18]. Likewise, such particularity supports beneficial healing effects on other avascular tissues, such as tendons, i.e., advanced angiogenesis and advanced healing of the transected Achilles’s tendon, and regeneration of transected or crushed skeletal muscle [13]. Similarly, its role in reversing liver cirrhosis and counteracting portal hypertension further confirms BPC 157’s capacity to suppress pathological angiogenesis [3]. Second, BPC 157 therapy implements Folkman’s cornea concept (cornea neovascularization goes with tumor growth; inhibited corneal neovascularization, inhibited tumor growth) [7,8,9], and exhibits a particular anti-tumor effect in the human melanoma cell line, it inhibits the VEGF effect [16], attributed to controlling the VEGF system as well [16]. In vivo, in mice, BPC 157 therapy considerably reduced the number of lung metastases induced by melanoma B-16 (unpublished). Furthermore, in mice with C26 colon adenocarcinoma, BPC 157 counteracted tumor cachexia, severe muscle wasting, corrected deranged muscle proliferation, and myogenesis, counteracted weight loss, and markedly prolonged survival [15]. BPC 157 significantly counteracted an increase in proinflammatory and procachectic cytokines such as interleukin 6 (IL-6) and tumor necrosis factor (TNF-alpha) [15]. Third, all these points may be supported by its special interaction with various molecular pathways (i.e., BPC 157 counteracted leaky gut syndrome) [19], especially with the NO-system [11,20,21,22] as a whole. Mainly, this was done by application of L-NAME, L-arginine, given alone and together (L-NAME+L-arginine) (NO-agents triple-application) to oversee the effect on NO-system function as a whole, inhibition (L-NAME), overstimulation (L-arginine), and immobilization (L-NAME+L-arginine). This implies a wide therapy counteracting and controlling potential as follows:

(i) NO-release (i.e., BPC 157 strongly opposes the NO-over-release induced by L-arginine) [11], and whatever the effect on NO, and eNOS gene expression, increase or decrease, it is always combined with the counteraction of free radicals formation) [11];
(ii) NO-synthase (NOS)-inhibition (L-NAME) (counteracted hypertension, and pro-coagulant effect) [11];
(iii) NOS-over-activity (L-arginine) (counteracted hypotension and anti-coagulant effect) [11];
(iv) NO-system immobilization (L-NAME+L-arginine) (reversal) [11].

Thus, there is a therapy effect i.e., in hypertension, hypotension, and maintained thrombocyte function (without affecting coagulation cascade) [11], signaling pathways controlling vasomotor tone [20,21,22] (VEGFR2-Akt-eNOS and Src-Caveolin-1-eNOS), thus, a modulatory effect in well-orchestrated function in NO-system relation. Consistently, BPC 157 does not induce uncontrolled NO-increase; the effect on NO, increase or decrease, is always combined with counteraction of free radicals formation [11]. As a generalization, measurement of lipid peroxidation was carried out using one of the most commonly employed methods in biomedical research for lipid peroxidation, the thiobarbituric acid reactive substances (TBARS) assay, expressed as malondialdehyde (MDA) equivalents. As a further generalization, utilizing a triple-application approach involving L-NAME, L-arginine, and their combination [11], the review [11] summarizes over 80 distinctive targets and numerous variations in NO-system responses at those sites. Notably, many experimental data exemplify support for this protective balance. For example, the increased MDA-values and the decreased NO-values, BPC 157 reversed to the normal healthy values (perforation, severe ischemic/reperfusion colitis) [23,24,25,26]. Counteraction of the increased NO and MDA levels occurred with vessel occlusion, cirrhosis, cytostatic application, and haloperidol application [26,27,28,29,30,31,32].

Naturally, clarifying how this selectivity is achieved mechanistically would strengthen the argument. Furthermore, as the indicative clues, but not definitive answers, long before angiogenesis begins, BPC 157 therapy rapidly activates collateral blood vessels [3,4,5,6]. These occurred in reversing thrombosis and Virchow triad circumstances in counteraction of the severe multiorgan and vascular failure in occlusion/occlusion-like syndromes [3,4,5,6]. Illustratively, for counteraction, there was the azygos vein direct blood flow delivery, providing the reestablishment of the reorganized blood flow, and full rescue was achieved [3,4,5,6]. Note, portal hypertension (a known insight of pathological angiogenesis), along with caval and intracranial hypertension and aortal hypotension, was regularly eliminated/attenuated [3,4,5,6]. Furthermore, Fourier transform infrared spectroscopy evidenced in the vessel wall, within minutes, a rapid change in the lipid contents and protein secondary structure conformation, produced instantly via BPC 157 therapy [33]. This shows supporting the vessel function even in the worst circumstances.

With these caveats, these BPC 157 effects (i.e., advanced healing) control a balance between competing proangiogenic and antiangiogenic mediators and the NO-system [17,18]. This would be done within the implementation of the updated cytoprotection concept (innate epithelial and endothelial cell protection, long ago postulated in the stomach [2,3]) by targeting angiogenesis and NO’s cytotoxic and damaging actions, but maintaining, promoting, or recovering their essential protective functions [11]. Consequently, this key will show that such conceptual evidence and beneficial findings analysis, a large network of interconnected beneficial evidence supporting each other [2,3,4,5,6,11,12,13,14,15,16,17,18,19], fully support these particular relations and the essential controlling role of pentadecapeptide BPC 157 in angiogenesis and the NO-system function [11], and thereby, beneficial effects in neurodegenerative diseases [4,5,13,14] and anti-tumor potential [15,16].

In principle, such arguments should resolve the general concern of Józwiak and collaborators’ report [1] that the complexity and multifaceted nature of biological activity and interaction with multiple systems have provided unescapable risks of unanticipated adverse effects due to pleiotropic effects. Namely, with BPC 157 therapy, pleiotropic beneficial effects on the lesions in the brain, heart, lung, liver, kidney, and gastrointestinal tract, in the reversal of severe multiorgan and vessel failure, reversal of occlusion/occlusion-like syndrome as a whole [3,6], reversal of advanced Virchow triad circumstances, occurred without causing harm to other organs [2,3,4,5,6,11,12,13,14,15,16,17,18,19]. This sided with evidence that BPC 157 exhibited a negative limit test, 2 g/kg i.v. or i.g., without adverse effects in mice, and a lethal dose (LD1) was not achieved, a general lack of toxicity that would also show metabolites without harmful effect [2,3,4,5,6,11,12,13,14,15,16,17,18,19]. Furthermore, the scarce human studies so far done, regardless of all of the limitations in some of them (i.e., lacked a large sample size, ethnic variation, and sham control group) without adverse effects [34,35,36,37,38], encompassed a wide range of investigations (i.e., ulcerative colitis (double-blind phase II study) [34,35], knee pain [36], interstitial cystitis [37]). Together, this can be along with the large range of beneficial effects of the BPC 157 therapy indicated by the animal experiments.

## Figures and Tables

**Table 1:** Summarized predictions of Józwiak and collaborators’ review [1] on BPC 157 toxicity, speculation hypothesized on angiogenesis and NO-system dysregulation, over-stimulation of NO and endothelial nitric oxide synthase (eNOS), and complicated with vascular endothelial growth factor (VEGF), early growth response-1 (egr-1) gene, free radicals formation, tumorigenesis, and neurodegenerative diseases. Negative predictions were completely ruled out based on the evidence obtained in the studies and reviewed in reviews [2,3,4,5,6], evidencing targeting angiogenesis and NO’s cytotoxic and damaging actions, but maintaining, promoting, or recovering their essential protective functions.

Summarized Prediction of BPC 157 Toxicity Based on the Given Speculations Hypothesized in the Review of Józwiak and Collaborators [1]		Negative Prediction Was Ruled Out Based on the Evidence Obtained in the Studies, and Reviewed in Reviews
**1.**	**ANGIOGENESIS CONSEQUENCES**	BPC 157 therapy resolved cornea “angiogenic privilege”, and exhibited cornea healing, transparency maintenance, and beneficial effects on other tissues (i.e., tendon, muscle). These BPC 157 effects (i.e., advanced healing) control a balance between competing proangiogenic and antiangiogenic mediators.
1.1.	BPC 157→↑angiogenesis→carcinoma	BPC 157 strongly opposed corneal neovascularization. BPC 157 lacks the first false wrong interconnections (i.e., Folkman: angiogenic bioactivity of a given molecule→↑cornea neovascularization→↑carcinoma), but has the second positive interconnections (i.e., Folkman: bioactivity of a given molecule→↓cornea neovascularization→↓carcinoma). Inhibiting corneal neovascularization, and all other particular healing effects of BPC 157 on angiogenesis in other tissues (advanced: tendon, muscle vs. inhibited: pathological angiogenesis in cirrhosis, portal hypertension), including noted anti-tumor effects, could suggest a particular anti-tumor effect by BPC 157 therapy.
1.2.	BPC 157→↑VGEF→carcinoma	In damaged muscle and tendon, reflecting achieved healing already at earlier points, advanced healing by BPC 157 therapy increased VEGF expression in the first days while subsequently decreasing in later days. BPC 157 counteracted the effects of VGEF and inhibited cell growth and VEGF signaling via the MAPK kinase pathway in the human melanoma cell line.
1.3.	BPC 157→↑NO→↑free radicals→carcinoma	BPC 157 strongly opposes the NO-over-release induced by L-arginine and counteracts free radical formation. In various injury models, BPC 157 therapy increases or decreases NO levels and eNOS gene expression, but always decreases free radical formation.
1.4.	BPC 157→↑eNOS→↑free radicals→carcinoma	
1.5.	BPC 157→↑egr-1→atherosclerosis	BPC 157, simultaneously with stimulated expression of egr-1 gene, induced increased expression of its repressor nab2 gene. BPC 157 therapy immediately established a negative feedback loop between egr-1 and nab2, which can be a particular key in the prompt healing effect of BPC 157 therapy. Besides, the beneficial effects of BPC 157 therapy fully involve recovery of the implicated pathology (i.e., cardiovascular, liver, and brain).
1.6.	BPC 157→↑egr-1→stenosed calcific valvular disease	
1.7.	BPC 157→↑egr-1→cardiac hypertrophy	
1.8.	BPC 157→↑egr-1→cerebral ischemia	
1.9.	BPC 157→↑egr-1→carcinoma	
**2.**	**HARM OF BPC 157 METABOLITES**	BPC 157 exhibited a negative limit test, 2 g/kg i.v. or i.g., without adverse effects in mice, and a lethal dose (LD1) was not achieved.
2.1.	BPC 157→↑metabolite proline→↑superoxide formation	BPC 157 strongly counteracted the formation of free radicals.
**3.**	**BPC 157 STIMULATORY EFFECT** **ON THE NO-SYSTEM**	BPC 157 strongly opposed the NO-over-release induced by L-arginine and counteracted free radicals formation. BPC 157 therapy consistently counteracted Parkinson’s disease and Alzheimer’s disease animal models.
3.1.	BPC 157→↑NO→↑free radicals→Parkinson’s disease	
3.2.	BPC 157→↑NO→↑free radicals→Alzheimer’s disease	
4.	**GENERAL CONCERN**	BPC 157 therapy: targeting angiogenesis and NO’s cytotoxic and damaging actions, but maintaining, promoting, or recovering their essential protective functions
4.1.	Complexity and multifaceted nature of BPC 157’s biological activity, its interaction with multiple systems in the body→↑unescapable risks of unanticipated adverse effects of BPC 157 therapy due to its pleiotropic effects	In severe multiorgan and vessel failure, occlusion/occlusion-like syndrome, BPC 157 therapy effects could resolve even the essential issue (i.e., Virchow triad circumstances, hemorrhage, and thrombosis). They are safe concerning the cure of each organ involved, brain, heart, lung, liver, kidney, and gastrointestinal tract, and do not produce any adverse effect on account of their beneficial effects.
**5.**	**HUMAN DATA**	A wide range of investigations (i.e., ulcerative colitis, knee pain, interstitial cystitis) aligns with the large range of the beneficial effects of the BPC 157 therapy indicated by the animal experiments.
5.1.	Scarcity of human data	Human evidence was improved with the clinical studies (phase I and phase II) that Józwiak and collaborators [1] missed.
**6.**	**SPECIFIC CONCERN, UNCERTAIN ANIMAL DATA TRANSLATION, NOT RELIABLE RESULTS**	Ulcerative colitis, knee pain, and interstitial cystitis in patients taken together, align with the large range of the beneficial effects of the BPC 157 therapy indicated by the animal experiments.
6.1.	Uncertain transfer of animal data, not reliable results due to a lack of comparison of different methods of drug administration in one model, such as oral vs. intraperitoneal and others.	Making the results more reliable is well done with the comparison of different methods of drug administration in one model, such as oral vs. intraperitoneal and others. This includes intraperitoneal vs. per-oral, thin layer of the cream at the site of injury vs. intraperitoneal, topical at injured nerve, intraperitoneal, intragastric, intraperitoneal vs. intragastric vs. intrarectal, intramuscular vs. percutaneous into the bone defect, intramuscular vs. intragastric, topical application at the brain vs. intraperitoneal vs. intragastric, eye drops vs. intraperitoneal vs. per-oral, and eye drops vs. intraperitoneal.

## References

[1] Józwiak M., Bauer M., Kamysz W., Kleczkowska P. (2025). Multifunctionality and Possible Medical Application of the BPC 157 Peptide—Literature and Patent Review. Pharmaceuticals.

[2] Seiwerth S., Rucman R., Turkovic B., Sever M., Klicek R., Radic B., Drmic D., Stupnisek M., Misic M., Vuletic L.B. (2018). BPC 157 and standard angiogenic growth factors. Gastrointestinal tract healing, lessons from tendon, ligament, muscle and bone healing. Curr. Pharm. Des..

[3] Sikiric P., Sever M., Krezic I., Vranes H., Kalogjera L., Smoday I.M., Vukovic V., Oroz K., Coric L., Skoro M. (2024). New studies with stable gastric pentadecapeptide protecting gastrointestinal tract. Significance of counteraction of vascular and multiorgan failure of occlusion/occlusion-like syndrome in cytoprotection/organoprotection. Inflammopharmacology.

[4] Sikiric P., Boban Blagaic A., Strbe S., Beketic Oreskovic L., Oreskovic I., Sikiric S., Staresinic M., Sever M., Kokot A., Jurjevic I. (2024). The stable gastric pentadecapeptide BPC 157 pleiotropic beneficial activity and its possible relations with neurotransmitter activity. Pharmaceuticals.

[5] Sikiric P., Gojkovic S., Krezic I., Smoday I.M., Kalogjera L., Zizek H., Oroz K., Vranes H., Vukovic V., Labidi M. (2023). Stable gastric pentadecapeptide BPC 157 may recover brain-gut axis and gut-brain axis function. Pharmaceuticals.

[6] Sikiric P., Gojkovic S., Knezevic M., Tepes M., Strbe S., Vukojevic J., Duzel A., Kralj T., Krezic I., Zizek H. (2023). Stable Gastric Pentadecapeptide BPC 157: Prompt particular activation of collateral pathways. Curr. Med. Chem..

[7] Hanahan D., Weinberg R.A. (2008). Retrospective: Judah Folkman (1933–2008). Science.

[8] Folkman J. (1971). Tumor angiogenesis: Therapeutic implications. N. Engl. J. Med..

[9] Folkman J., Klagsbrun M. (1987). Angiogenic factors. Science.

[10] Moncada S. (1994). Nitric oxide. J. Hypertens. Suppl..

[11] Sikiric P., Drmic D., Boban Blagaic A., Tvrdeic A., Krezic I., Gojkovic S., Zizek H., Sikiric S., Strbe S., Smoday I.M., Ray A., Gulati K. (2023). Stable gastric pentadecapeptide BPC 157 and NO-system. Nitric Oxide: From Research to Therapeutics.

[12] Seiwerth S., Milavic M., Vukojevic J., Gojkovic S., Krezic I., Vuletic L.B., Pavlov K.H., Petrovic A., Sikiric S., Vranes H. (2021). Stable gastric pentadecapeptide BPC 157 and wound healing. Front. Pharmacol..

[13] Staresinic M., Japjec M., Vranes H., Prtoric A., Zizek H., Krezic I., Gojkovic S., Smoday I.M., Oroz K., Staresinic E. (2022). Stable gastric pentadecapeptide BPC 157 and striated, smooth, and heart muscle. Biomedicines.

[14] Sikiric P., Udovicic M., Barisic I., Balenovic D., Zivanovic Posilovic G., Strinic D., Uzun S., Sikiric S., Krezic I., Zizek H. (2022). Stable gastric pentadecapeptide BPC157 as useful cytoprotective peptide therapy in the heart disturbances, myocardial infarction, pulmonary hypertension, arrhythmias, and thrombosis presentation. Biomedicines.

[15] Kang E.A., Han Y.M., An J.M., Park Y.J., Sikiric P., Kim D.H., Kwon K.A., Kim Y.J., Yang D., Tchah H. (2018). BPC157 as potential agent rescuing from cancer cachexia. Curr. Pharm. Des..

[16] Radeljak S., Seiwerth S., Sikiric P. (2004). BPC 157 inhibits cell growth and VEGF signalling via the MAPK kinase pathway in the human melanoma cell line. Melanoma Res..

[17] Masnec S., Kokot A., Zlatar M., Kalauz M., Kunjko K., Radic B., Klicek R., Drmic D., Lazic R., Brcic L. (2015). Perforating corneal injury in rat and pentadecapeptide BPC 157. Exp. Eye Res..

[18] Sikiric P., Kokot A., Kralj T., Zlatar M., Masnec S., Lazic R., Loncaric K., Oroz K., Sablic M., Boljesic M. (2023). Stable gastric pentadecapeptide BPC 157—Possible novel therapy of glaucoma and other ocular conditions. Pharmaceuticals.

[19] Park J.M., Lee H.J., Sikiric P., Hahm K.B. (2020). BPC 157 rescued NSAID-cytotoxicity via stabilizing intestinal permeability and enhancing cytoprotection. Curr. Pharm. Des..

[20] Wu H., Wei M., Li N., Lu Q., Shrestha S.M., Tan J., Zhang Z., Wu G., Shi R. (2020). Clopidogrel-induced gastric injury in rats is attenuated by stable gastric pentadecapeptide BPC 157. Drug Des. Devel. Ther..

[21] Hsieh M.J., Lee C.H., Chueh H.Y., Chang G.J., Huang H.Y., Lin Y., Pang J.S. (2020). Modulatory effects of BPC 157 on vasomotor tone and the activation of Src-Caveolin-1-endothelial nitric oxide synthase pathway. Sci. Rep..

[22] Hsieh M.J., Liu H.T., Wang C.N., Huang H.Y., Lin Y., Ko Y.S., Wang J.S., Chang V.H., Pang J.S. (2017). Therapeutic potential of pro-angiogenic BPC157 is associated with VEGFR2 activation and up-regulation. J. Mol. Med..

[23] Bilic Z., Gojkovic S., Kalogjera L., Krezic I., Malekinusic D., Knezevic M., Sever M., Lojo N., Kokot A., Kasnik K. (2021). Novel insight into Robert’s cytoprotection: Complex therapeutic effect of cytoprotective pentadecapeptide BPC 157 in rats with perforated stomach throughout modulation of nitric oxide-system. Comparison with L-arginine, ranitidine and pantoprazole therapy and L-N^G^-nitro-L-arginine methyl ester worsening. J. Physiol. Pharmacol..

[24] Drmic D., Samara M., Vidovic T., Malekinusic D., Antunovic M., Vrdoljak B., Ruzman J., Milkovic Perisa M., Horvat Pavlov K., Jeyakumar J. (2018). Counteraction of perforated cecum lesions in rats: Effects of pentadecapeptide BPC 157, L-NAME and L-arginine. World J. Gastroenterol..

[25] Amic F., Drmic D., Bilic Z., Krezic I., Zizek H., Peklic M., Klicek R., Pajtak A., Amic E., Vidovic T. (2018). Bypassing major venous occlusion and duodenal lesions in rats, and therapy with the stable gastric pentadecapeptide BPC 157, L-NAME and L-arginine. World J. Gastroenterol..

[26] Duzel A., Vlainic J., Antunovic M., Malekinusic D., Vrdoljak B., Samara M., Gojkovic S., Krezic I., Vidovic T., Bilic Z. (2017). Stable gastric pentadecapeptide BPC 157 in the treatment of colitis and ischemia and reperfusion in rats: New insights. World J. Gastroenterol..

[27] Sever A.Z., Sever M., Vidovic T., Lojo N., Kolenc D., Vuletic L.B., Drmic D., Kokot A., Zoricic I., Coric M. (2019). Stable gastric pentadecapeptide BPC 157 in the therapy of the rats with bile duct ligation. Eur. J. Pharmacol..

[28] Luetic K., Sucic M., Vlainic J., Halle Z.B., Strinic D., Vidovic T., Luetic F., Marusic M., Gulic S., Pavelic T.T. (2017). Cyclophosphamide induced stomach and duodenal lesions as a NO-system disturbance in rats: L-NAME, L-arginine, stable gastric pentadecapeptide BPC 157. Inflammopharmacology.

[29] Sucic M., Luetic K., Jandric I., Drmic D., Sever A.Z., Vuletic L.B., Halle Z.B., Strinic D., Kokot A., Seiwerth R.S. (2019). Therapy of the rat hemorrhagic cystitis induced by cyclophosphamide. Stable gastric pentadecapeptide BPC 157, L-arginine, L-NAME. Eur. J. Pharmacol..

[30] Gojkovic S., Krezic I., Vrdoljak B., Malekinusic D., Barisic I., Petrovic A., Horvat Pavlov K., Kolovrat M., Duzel A., Knezevic M. (2020). Pentadecapeptide BPC 157 resolves suprahepatic occlusion of the inferior caval vein, Budd-Chiari syndrome model in rats. World J. Gastrointest. Pathophysiol..

[31] Kolovrat M., Gojkovic S., Krezic I., Malekinusic D., Vrdoljak B., Kasnik Kovac K., Kralj T., Drmic D., Barisic I., Horvat Pavlov K. (2020). Pentadecapeptide BPC 157 resolves Pringle maneuver in rats.; both ischemia and reperfusion. World. J. Hepatol..

[32] Belosic Halle Z., Vlainic J., Drmic D., Strinic D., Luetic K., Sucic M., Medvidovic-Grubisic M., Pavelic Turudic T., Petrovic I., Seiwerth S. (2017). Class side effects: Decreased pressure in the lower oesophageal and the pyloric sphincters after the administration of dopamine antagonists, neuroleptics, anti-emetics, L-NAME, pentadecapeptide BPC 157 and L-arginine. Inflammopharmacology.

[33] Gamulin O., Oroz K., Coric L., Krajacic M., Skrabic M., Dretar V., Strbe S., Talapko J., Juzbasic M., Krezic I. (2022). Fourier transform infrared spectroscopy reveals molecular changes in blood vessels of rats treated with pentadecapeptide BPC 157. Biomedicines.

[34] Ruenzi M., Stolte M., Veljaca M., Oreskovic K., Peterson J., Ulcerative Colitis Study Group (2005). A multicenter, randomized, double blind, placebo-controlled phase II study of PL 14736 enema in the treatment of mild-to-moderate ulcerative colitis. Gastroenterology.

[35] Veljaca M., Pavic Sladoljev D., Mildner B., Brajsa K., Bubenik M., Stipanicic S., Parnham M. (2003). Safety, tolerability and pharmacokinetics of PL14736, a novel agent for treatment of ulcerative colitis, in healthy male volunteers. Gut.

[36] Lee E., Padgett B. (2021). Intra-articular injection of BPC 157 for multiple types of knee pain. Altern. Ther. Health Med..

[37] Lee E., Walker C., Ayadi B. (2024). Effect of BPC-157 on symptoms in patients with interstitial cystitis: A pilot study. Altern. Ther. Health Med..

[38] Lee E., Burgess K. (2025). Safety of intravenous infusion of BPC157 in humans: A pilot study. Altern. Ther. Health Med..

